# Stochastic neutral modelling of the Gut Microbiota’s relative species abundance from next generation sequencing data

**DOI:** 10.1186/s12859-015-0858-8

**Published:** 2016-01-20

**Authors:** Claudia Sala, Silvia Vitali, Enrico Giampieri, Ìtalo Faria do Valle, Daniel Remondini, Paolo Garagnani, Matteo Bersanelli, Ettore Mosca, Luciano Milanesi, Gastone Castellani

**Affiliations:** 1 Department of Physics and Astronomy, University of Bologna, Bologna, 40127 Italy; 2 CAPES Foundation, Ministry of Education of Brazil, Brasìlia, DF, 70040-020 Brazil; 3 Department of Experimental, Diagnostic and Specialty Medicine, University of Bologna, Bologna, 40138 Italy; 4 Institute of Biomedical Technologies, National Research Council, Milan, 20090 Segrate Italy

**Keywords:** Microbiota, 16S RNA, OTU, RSA, Biodiversity, Ecological modelling

## Abstract

**Background:**

Interest in understanding the mechanisms that lead to a particular composition of the Gut Microbiota is highly increasing, due to the relationship between this ecosystem and the host health state. Particularly relevant is the study of the Relative Species Abundance (RSA) distribution, that is a component of biodiversity and measures the number of species having a given number of individuals. It is the universal behaviour of RSA that induced many ecologists to look for theoretical explanations. In particular, a simple stochastic neutral model was proposed by Volkov et al. relying on population dynamics and was proved to fit the coral-reefs and rain forests RSA. Our aim is to ascertain if this model also describes the Microbiota RSA and if it can help in explaining the Microbiota plasticity.

**Results:**

We analyzed 16S rRNA sequencing data sampled from the Microbiota of three different animal species by Jeraldo et al. Through a clustering procedure (UCLUST), we built the Operational Taxonomic Units. These correspond to bacterial species considered at a given phylogenetic level defined by the similarity threshold used in the clustering procedure. The RSAs, plotted in the form of Preston plot, were fitted with Volkov’s model. The model fits well the Microbiota RSA, except in the tail region, that shows a deviation from the neutrality assumption. Looking at the model parameters we were able to discriminate between different animal species, giving also a biological explanation. Moreover, the biodiversity estimator obtained by Volkov’s model also differentiates the animal species and is in good agreement with the first and second order Hill’s numbers, that are common evenness indexes simply based on the fraction of individuals per species.

**Conclusions:**

We conclude that the neutrality assumption is a good approximation for the Microbiota dynamics and the observation that Volkov’s model works for this ecosystem is a further proof of the RSA universality. Moreover, the ability to separate different animals with the model parameters and biodiversity number are promising results if we think about future applications on human data, in which the Microbiota composition and biodiversity are in close relationships with a variety of diseases and life-styles.

## Background

The Gut Microbiota (GM) [1] is found in virtually any Metazoan, from invertebrates to vertebrates. The human body, for example, is home to roughly 10 times more microbial than human cells. The collective genome of these symbiotic microorganisms (called Microbiome) [2] constantly interacts with the host genome, forming the so called Metagenome [3]. As a result of millions of years of coevolution, the microbial genome stands in dynamical relationship with the host organism and helps it in crucial functions. These include metabolic processes like food absorption and short chain fatty Acid (SCFA) and vitamins production [4], but also the shaping, control and protection of the immune system development [5]. It is through the interaction between the different components of the Metagenome that the host ‘health phenotype’ is defined [6, 7]. The Microbiome, and especially the Gut Microbiome (GM) is linked, through an interdependence relationship, to the host immune system [8] and metabolism [9] and it is crucial for a large number of physio-pathological conditions and diseases. These include inflammatory and metabolic diseases [9], such as Obesity [10], Metabolic Syndrome and Type 2 Diabetes [11]. In addition GM composition is influencing Ageing [12]. Moreover, recent advances in sequencing methods have led to new knowledge about the role of GM also on Brain development and neural disorders such as Autism [13] and Multiple Sclerosis [14]. The bidirectional cross-talk between host and GM is supported by GM transplants results (e.g. induction of donor phenotypes in the host) [10] and by the association of GM composition with pathological states [6]. Thus, GM is sensitive to environmental stimuli (particularly to nutrition), having an high individual specificity and plasticity and being modifiable by pharmacological agents such as pre- and pro-biotics, antibiotics and GM transplants. One of the main characteristics of Metagenome, that is crucial in the case of unhealthy people, is the molecular composition of the intersection between the host and the Microbiome. This interface is the way by which the host and the Microbiota communicate. Such interaction is bidirectional and history-dependent and can be characterized as a function of the exchanged metabolic, genetic and immunological bio-molecules [15].

GM is a complex ecosystem with a complex dynamics, that derives from the interactions with the host diet, life-style and health state, but also with components such as the virome (the set of viruses in the host body) and the Immune System [16]. Nowadays, the availability of Next Generation Sequencing Methods (NGS), for the characterization of bacterial communities, contributed to the creation of a new research field, called Metagenomics. Metagenomics is the set of omics measurements that quantify the composition of the metagenome and the interactions between the host and the microbiome at multiple levels: DNA (metagenome), RNA (meta-transcriptome), protein (meta-proteome) and metabolic network (metabolome). Classical microbiology largely relied on clonal culture techniques and for this reason turned out to be biased. In the 1990s, it has been estimated that the currently cultivable microorganisms represented only a small fraction (less than 1 *%*) of the total microbes within a given habitat [7], proving that culture methods were not able to access the vast majority of bacterial organisms. The great benefit of Metagenomics is indeed to overcome such issue. Metagenomics allows to sample the whole bacterial population, without the need of culturing and without the need of knowing in advance which microorganism to look for, as required for example by microarrays. This purpose is commonly achieved by sequencing the 16S rRNA gene. This choice is due to the fact that the 16S rRNA is highly conserved between different species of bacteria and, for this reason, delivers informations about their evolutionary relationships. Through 16S rRNA sequencing, in particular, one can sample bacteria in a community obtaining their phylogenetic relationships, and this turns out to be particularly useful if we want to give an ecological description of GM.

The quantification of biodiversity and the modelling of the dynamics that brings to a certain composition of GM are important tasks for the assessment of the homeostasis degree and the ‘carrying capacity’ of ecosystems, as well as for the prediction of their evolution, and, for this purpose, mathematical models result pretty useful [17]. Here we propose a new method to describe the GM dynamics from an ecological point of view. We focus on the GM biodiversity, and in particular on one of its components: the Relative Species Abundance distribution (RSA). This is defined within a single phylogenetic level and refers to how common or rare a species is relative to other species. Interestingly, ecologists are widely fascinated by the ecosystems RSA, and the reason for this is that it follows very similar patterns over a wide range of ecological communities [18]: in a diverse array of populations, ranging from an open-ocean planktonic copepod community, to a tropical bats community, but also in a community of rainforest trees and of British breeding birds, the Relative Species Abundances are recognizably drawn from a single family of distributions, ranging from the Log-Series [19] to a highly skewed and unveiled Log-Normal [20]. Early attempts to fit observed data with statistical distributions were mainly inductive approaches. Two milestones among these are indeed Fisher’s Log-Series [19]and Preston’s Log-Normal [20]. Starting from the hypothesis that the number of collected individuals belonging to a given species follows a Poisson distribution, Fisher showed that the number of different organisms observed in a single sample, if the different species are not equally abundant, can be described by a Negative Binomial distribution. Then he eliminated the zero abundance class (species too rare to be sampled) and assumed that the total number of species in a community was infinite. Finally he obtained a Log-Series distribution that was able to fit different datasets. A few years later, Preston criticized the Log-Series on the grounds that it was not a good fit to the data that he had assembled. Preston argued that RSA distributions were actually Log-Normal, partly due to the Central Limit Theorem, and that the Log-Series resulted from under-sampling. Later, several attempts were made in order to derive a theory of RSA, that was based on hypotheses about how ecological communities are organized, as reviewed in [18]. Here we just note that these theories present many shortcomings, among which a not entirely exhaustive biological interpretation, the presence of many parameters, and, besides that, the inability to explain real data. In this work, we would like to give an explanation for the GM RSA distribution relying on population dynamics. We would like our model to fit experimental data in both situations observed by Fisher and Preston (i.e. Log-Series and Log-Normal like RSA) and we also would like our model to be simple, without too many parameters and to have a clear biological interpretation. For this purpose, we based our work on the ecological neutral theory.

Modern ecological theories can be distinguished in essentially two main schools of thought: the niche assembly perspective and the dispersal one [18]. The niche assembly perspective holds that communities are groups of interacting species whose presence or absence and even their relative abundance can be deduced from deterministic ‘assembly rules’ that are based on the ecological niches or functional roles of each species. Here, the concept of ‘ecological niche’ summarizes the interactions between species and their environment, and is thus defined by two components: the requirement for an organism of a given species to live in a given environment (the extent to which a limiting factor influences the birth and death rate of that species) and the impact of the species on its environment (the extent to which the growth of a population alters the limiting factor, i.e. the availability of a resource or the density of a predator or parasite). According to this view, species coexist in interactive equilibrium and a stable co-existence among competing species is made possible by niche partitioning [21]. The other world view is the dispersal assembly perspective, which asserts that communities are open, non-equilibrium assemblages of species largely thrown together by chance, history, and random dispersal. Species come and go, their presence or absence is dictated by random dispersal and stochastic local extinction. Actually we will refer to a particular class of dispersal theories, those called ‘neutral’, in which ecological communities are structured entirely by ecological drift (i.e. demographic stochasticity), random migration, and random speciation. By neutral we mean that the theory treats organisms in a trophically defined community as essentially identical in their per capita probabilities of giving birth, dying, migrating, and speciating (ecological equivalence) [18].

In particular, in this work we show that the GM RSA can be well described by the simple stochastic neutral model initially proposed by [22, 23] for the coral-reefs and tropical trees ecosystems. This model neglects species interaction and assumes that the number of individuals within a species could change only because of a birth or a death. To this birth-death process a further constant influx term is added. This term includes phenomena such as immigration or speciation but can also be considered as the mean effect of species interactions. Moreover, the neutrality assumption supposes that all species are described by the same birth, death and influx parameters, keeping the model as simple as possible. The stationary distribution predicted by the model for the RSA is a Negative Binomial distribution. This has the peculiarity of including the Log-Series as particular case and of being able to fit also Log-Normal like data, hence is capable to describe both the common RSA shapes previously observed.

In the following, first we describe the GM data that we analysed. These include NGS data from [24], that involve the GM characterization of three different animal species: chicken, cattle rumen and swine. Starting from NGS data, we describe how we built empirical RSA distributions for the GM. In particular, the method includes a clustering procedure of the data sequences, in which changing the similarity threshold settings allows to consider the ecosystem at different phylogenetic levels. Then, we deepen the theoretical model by [22] and we show how the Negative Binomial distribution is obtained, also introducing the related biodiversity number, as proposed by Volkov [18, 22]. Finally, we show our results, according to which the model well fits the GM RSA and turns out to be capable to discriminate between the GM coming from different animal species. This is particularly clear if we compare the biodiversity numbers obtained for different animals and for different phylogenetic levels, that results to be consistent with the second order Hill’s number, a commonly used biodiversity index.

## Methods

We analysed the data from [24], available in the National Center for Biotechnology Information Sequence Reads Archive under the accession number [SRA052136.3]. This study was approved by the Institutional Animal Care and Use Committee of the University of Illinois and includes 16S rRNA sequencing data (454 Life Sciences pyrosequencing) from 5 animals: 1 chicken [SRA:SRR491179]; 2 cattle rumens [SRA:SRR491180, SRA:SRR491181]; and 2 swines [SRA:SRR491182, SRA:SRR491183]. Starting from the 16S rRNA sequences, we built the RSA distribution of the GUT ecological system computing the so called Operational Taxonomic Units (OTUs) through a clustering procedure based on sequence similarity. This was performed with four different similarity thresholds (90 *%*, 93 *%*, 95 *%* and 97 *%*), as explained below. In particular the RSA was obtained representing the OTUs abundances in the form of Preston plot [20]. This is the plot of how many species (y-axes) have a certain number of individuals (x-axis), with the x-axis transformed in logarithm to base 2 in order to compress the information of the otherwise very long tail of the distribution.

Before deepening the methodology, let us underline that in order to give an ecological description of the Microbiota, it is highly recommended to base the analysis on *de novo* OTUs, rather then on some taxonomic classification in which sequences are clustered through comparison with 16S rRNA databases such as RDP [25], Silva [26] or Greengenes [27]. In fact, although phylotype-based methods are appealing approaches, since they enable investigators to place labels onto sequences, indicating their relationships to previously cultured and characterized microbes, they are human-made methods and there are myriad examples of organisms that belong to the same species that have different phenotypes and organisms with the same phenotype belonging to different taxonomic lineages, without talking about unclassified organisms or about the fact that there are at least three different curated taxonomy outlines that contain significant conflicts with each other [28]. In order to estimate evolutionary relationships of organisms, genes, species, etc. the tool to be used is phylogenetic analysis, that is the analysis of OTUs [29, 30]. Basing our analysis on OTUs, we will not be able to give names to species, but we will have the great advantage of avoiding the loss of information due to a taxonomic classification.

With the aim of building OTUs, we clustered the 16S rRNA sequences with UCLUST [31]. UCLUST starts with an empty database in memory and then reads the sequences in input order. Thus, sequences were previously sorted by quality, since UCLUST is sequence order sensitive and needs the highest quality sequences at the beginning of the input file. The algorithm takes the first sequence as first seed, and then all other sequences are processed according to the following statement: if a sequence is similar to a seed within a fixed similarity threshold, then the query is assigned to its cluster; if a sequence is instead not similar to any seed, then it will become the seed for a new cluster. This means that the between classes distance will be at least (1 - similarity threshold), while the within class distance will be approximately maximized by such similarity threshold. Here, the similarity between two sequences is computed by USEARCH. The sequences are first globally aligned, then similarity is computed as the fraction of columns in the alignment that contain identical letters, while the distance between two sequences is given by (1 - similarity). In this counting, gaps were penalized according to default settings [31]. UCLUST has the drawback of producing clusters that will not have the similarity threshold as exact maximum distance inside them, but overcomes two of the main problems of sequencing analysis, that are time and memory costs. UCLUST is one of the most common sequences clustering algorithm, and the reason for that is its great advantage of needing to memorize just the seeds and to compare the query sequences just with such seeds.

We applied the UCLUST algorithm using four different similarity thresholds: 90 *%*, 93 *%*, 95 *%* and 97 *%*. In this way, because of the conservative feature of the 16S rRNA gene previously outlined, we obtained clusters (OTUs), that can be thought as groups of bacteria of the same taxon at a particular phylogenetic level [28], for four different levels. Here, the similarity threshold is what defines the phylogenetic level at which we compute the RSA, the scale level at which we study the ecosystem.

Starting from UCLUST results, we estimated the OTUs abundances, that correspond to species abundances. We then filtered for singletons (OTUs with just one sequence inside) in order to minimize the inclusion of sequencing artefacts [32]. Since the absence of singletons violates some fundamental assumptions of species richness analysis, we randomly removed one sequence per OTU, causing all OTUs with originally two reads (doubletons) to become singletons, those with three reads to become doubletons, and so on. The effect of an even more conservative interpretation of 454 reads on species richness analysis was evaluated by consecutively omitting doubletons and tripletons from the initial data sets. Thus, we computed the RSA in the form of Preston plot for all four data sets: singletons retained, singletons excluded, doubletons excluded and tripletons excluded. The greatest difference appeared between retaining or removing singletons from the data set, thus, considering the less restrictive case, we chose to exclude just singletons, and to subtract 1 from all the other OTUs abundances. Note that, even if the singleton removal is supposed to substantially reduce RSA errors due to the sequencing procedure, the error on the reconstruction of the empirical RSA, due to all the other experimental steps (starting from the sampling itself) is not valuable since we have just one GM sample for animal. However, the fact that we find consistent results for the couples of animals of the same species, as showed later, suggests that such error can be neglected.

The GM RSA was modelled according to the neutral theory of ecosystems proposed by [22]. Here, the dynamics of the population of a single species are governed by generalized birth and death events, that include speciation, immigration and emigration. If we neglect inter-species interactions after the community has reached a steady state, the number of individuals *n* of a given species evolves according to equation

(1) $$ \frac{dn}{dt} = b \cdot n - d \cdot n + S,   $$

where *b* and *d* denote the per-capita density-independent birth and death rates, while the presence of the constant influx *S* produces a density dependence effect, which causes a rare species advantage and which can arise due to effective rates of immigration, emigration, speciation or extinction in a local community, but can also be due to intraspecific interactions. Note that because of the species equivalence assumption, this equation actually holds for all the GM species.

In order to take into account stochasticity, Eq. 1 should be rewritten in a probabilistic form, as accomplished by the Chemical Master Equation [33, 34]

(2) $$ \frac{\partial P_{n}(t)}{\partial t}=P_{n-1}(t)b_{n-1}+P_{n+1}(t)d_{n+1}-P_{n}(t)(b_{n}+d_{n})   $$

Here the influx *S* has been included in the birth term, setting

(3) $$ b_{n} = b \cdot (n + \Upsilon),  $$

where *Υ*=*S*/*b*, while the death rate simply is

(4) $$ d_{n} = d \cdot n.  $$

The stationary solution is easily obtained exploiting the current probability conservation condition (detailed balance):

(5) $$ P_{n} = P_{0} \prod_{i=0}^{n-1} \frac{b_{i}}{d_{i+1}} = P_{0} \prod_{i=0}^{n-1} \frac{b \cdot (i+S/b)}{d \cdot (i+1)}.  $$

Here we consider *n*>0 and we can deduce *P*_0_ from the normalization condition $\sum _{n\geq 0} P_{n} = 1$. The stationary solution turns out to be a Negative Binomial distribution

(6) $$ P_{n} = \frac{(1-b/d)^{S/b}}{\Gamma(S/b)} \frac{(b/d)^{n}}{n!} \Gamma(n + S/b).   $$

with two parameters: *b*/*d* and *S*/*b*.

The average number of species with *n* individuals, given the total number of species N, is

(7) $$ \phi_{n} = N \cdot P_{n}.  $$

Since the zero abundance class can not be observed, the mean number of observed species is

(8) $$ N_{obs} = N-\phi_{0} = N - N \cdot (1-b/d)^{S/b},  $$

and the average number of species actually in the community, that is the RSA normalization factor, is

(9) $$ N = \frac{N_{obs}}{1-(1-b/d)^{S/b}},  $$

from which

(10) $${} {\fontsize{9.2pt}{9.6pt}{\begin{aligned} P_{RSA}(n) = \frac{N_{obs}}{1-(1-b/d)^{S/b}} \frac{(1-b/d)^{S/b}}{\Gamma(S/b)}\frac{(b/d)^{n}}{n!}\Gamma(n+S/b), \end{aligned}}}   $$

where

(11) $${} {\fontsize{8.8pt}{9.6pt}{\begin{aligned} \theta = \frac{N_{obs}(1-b/d)^{S/b}}{\left[1-(1-b/d)^{S/b}\right]\Gamma(S/b)} = \frac{N_{obs}}{\left[(1-b/d)^{-S/b}-1\right]\Gamma(S/b)} \end{aligned}}}   $$

is the Hubbell biodiversity number [18], as illustrated in Volkov’s work [22].

## Results and Discussion

**Fig. 1:**
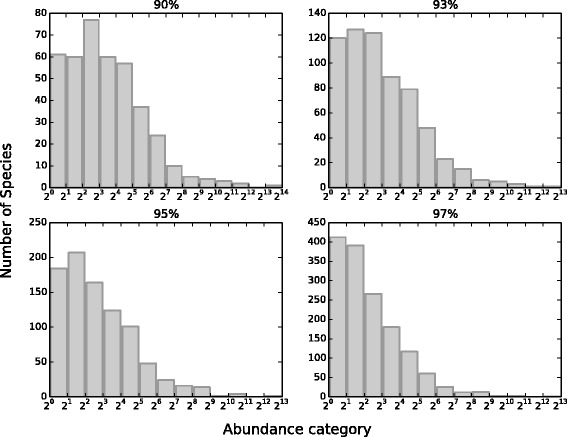
Preston plot of one cattle rumen sample. Empirical RSAs have been built considering four different similarity threshold: 90 *%*, 93 *%*, 95 *%* and 97 *%*. The RSA tends to become more Log-Series like for higher similarity thresholds, that correspond to finer phylogenetic levels

Plotting the Microbiota RSA distribution in the form of Preston plot revealed a first interesting behaviour of this ecosystem. In fact, Fig. 1 shows that if we consider different phylogenetic levels, that is we build the OTUs using different similarity thresholds, we obtain RSA distributions with diverse shapes. More exactly, with a threshold of 97 *%*, the RSA almost resembles a Log-Series distribution, while shifting towards lower similarity percentages, the RSA looks always more Log-Normal like. Actually, a similar trend of the RSA have been observed in [22] for the coral-reef ecosystem, in which the authors firstly considered many small semi-isolated local communities and then assembled them into bigger and bigger metacommunities. So, it seems that considering the Gut Microbiota ecosystem at higher phylogenetic levels somehow corresponds, from a dynamical point of view, to assembling semi-isolated local communities into metacommunities. Moreover, in the past the RSA shape has been described in terms of Log-Series or Log-Normal distribution [18], but the Log-Series is a special case of the Negative Binomial, that is obtained when *S*/*b*→0 (see Eq. 6) [22], and, for what concerns the Log-Normal, it has been proved not to be an appropriate null model for the RSA [35], and its bell-shaped cases can be well described also by the Negative Binomial distribution, due to the fact that, dealing with experimental data, these two distributions can take on similar shapes and are often hard to distinguish in practice [36].

**Fig. 2:**
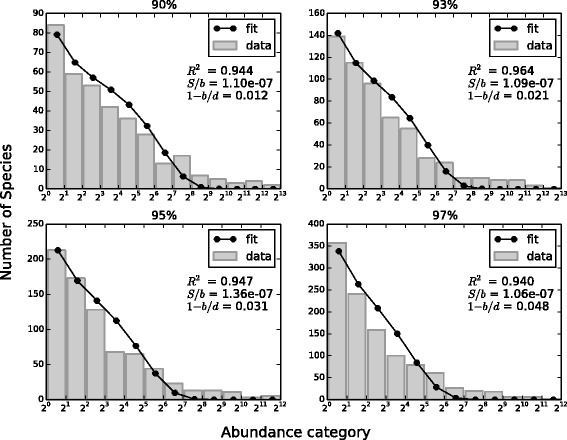
Fit of one swine’s RSA. Empirical RSA (*gray histogram*) and fit with Eq. 10 (*black line* and *dots*) relative to one swine sample for the four similarity thresholds considered

Our second analysis was to fit the GM empirical RSA with the Negative Binomial distribution 10. As already pointed out, because of its very long tail, the RSA is represented in the form of Preston plot, i.e. with a logarithm to base 2 x-axis. In this way, each bin actually contains the sum of the number of species with abundance category between its minimum and maximum (e.g. bin [2^2^,2^3^) represents the number of species having 4, 5, 6 or 7 individuals). Thus, the fit was computed considering the sum of the Negative Binomial distribution from the minimum to the maximum of each bin, that can be simply obtained subtracting the Negative Binomial cumulative in the minimum from the Negative Binomial cumulative in the maximum of the bin. Figure 2 shows the result for one swine sample. The model fits well the Gut Microbiota RSA and we obtained good R-squared (> 0.89) for all samples at all the similarity thresholds considered. Let us observe that, in truth, there is disagreement between the model and empirical data in the RSA tail, that is for high abundance categories. This suggests a deviation from the neutrality assumption. However, we can state that Volkov’s neutral model [22], that contemplates the simplest case in which species equivalence holds, is a good approximation of the empirical GM RSA.

**Fig. 3:**
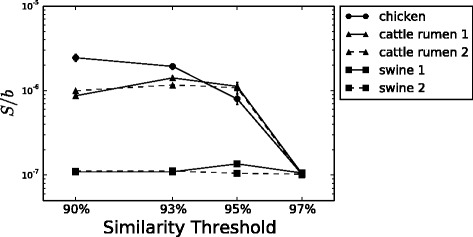
*S*/*b* versus similarity. Plot of the parameter *S*/*b* versus the similarity threshold used in computing the OTUs for all five samples. Note that *S*/*b* differentiate the three animal species considered and that it tends to diminish for high similarity thresholds, indicating that the RSA becomes more similar to a Log-Series, as attended from Fig. 1

After fitting the data with the Negative Binomial model, we exploited our result to assess if the model was able to discriminate between different animal species. We studied the variation of the parameter *S*/*b* with the similarity threshold, that is with the phylogenetic scale at which we observe the system, by plotting *S*/*b* versus the similarity threshold for each sample. Figure 3 shows the result. In general, for all samples *S*/*b* tends to diminish as the similarity threshold gets higher. This result is consistent with our qualitative description of the Preston plots, in which we assessed that the empirical RSA looked more Log-Series like for higher similarity thresholds (Fig. 1), in fact, as already pointed out, the Negative Binomial distribution tends to a Log-Series for *S*/*b*→0. Moreover, *S*/*b* results different for each animal group, especially at low similarity thresholds, and clusters together animals of the same species. This result is particularly interesting because of the biological meaning of *S*/*b*, or better of *S*. As exposed in the model description, this parameter is a constant influx term that takes into account effects such as immigration or speciation, but that can also be considered as a mean field effect of all external factors that influence the microbial community: diet, basal metabolic rate, health state, and so on.

**Fig. 4:**
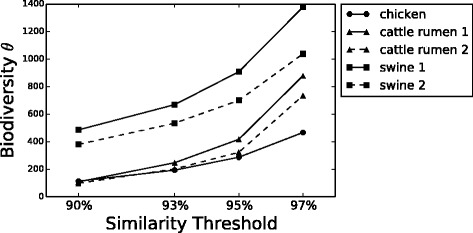
*θ* versus similarity. Hubbell biodiversity number *θ* [18, 22] computed with Eq. 11 for the five animals GM at the four similarity thresholds considered. Biodiversity increases with similarity and clusters animals according to their belonging species

**Fig. 5:**
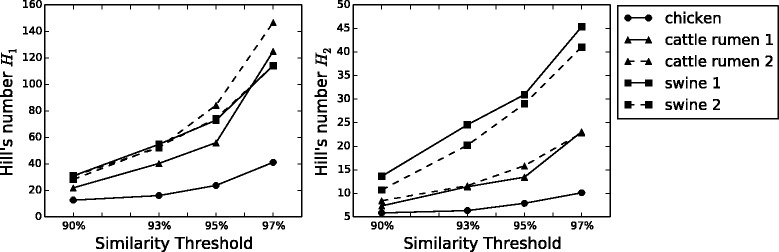
*H*_1_ and *H*_2_ versus similarity. First (*left*) and second (*right*) order Hill’s numbers for the five animals GM at the four similarity thresholds considered. The result is consistent with the biodiversity number *θ*

Finally, we computed the biodiversity number *θ* proposed by Volkov [22] through Eq. 11 using the fit parameters. Figure 4 shows how *θ* increases at higher similarity thresholds, pointing out that biodiversity increases at lower phylogenetic levels. Moreover, this parameter is capable to distinguish the animal species, exhibiting similar values and trends for animal of the same species and separating animal from different species. In order to compare our result with other commonly used biodiversity indexes, we computed, for all samples at the four similarity thresholds, the first and second order Hill’s numbers [37, 38]

(12) $$ H_{1} = e^{-{\sum_{i}^{N}}{p_{i} ln(p_{i})}}  $$

and

(13) $$ H_{2} = \frac{1}{{\sum_{i}^{N}}{{p_{i}^{2}}}}  $$

where *p*_*i*_ is the fraction of individuals in species *i* and *N* is the total number of species (OTUs). *H*_1_ and *H*_2_ are biodiversity estimators, that reach their maximum when every species has just one individual and the ecosystem has as many different species as possible, and shift towards their minima when all the individuals of the ecosystem belong to the same species. Figure 5 (left) and (right) show that also these diversity indexes discriminate between different animals; *H*_1_ separates just the chicken from the mammalians, but *H*_2_ separates all the three species, confirming that different animals Microbiota have different ecologies. Moreover, the biodiversity index *θ* computed with our modelling shows the same trend as Hill’s numbers, and in particular animal species are separated in exactly the same order as in *H*_2_. Comparing Fig. 4 with Fig. 5 it is clear that the different animals GM biodiversities estimated with the two methods have the same behavior.

## Conclusion

The Gut Microbiota behaves like an organ in the host organism and strictly interacts with that, participating to its homeostatic state, including health status. This is the reason for the growing interest in the dynamics that yield a particular GM composition. Here we showed that the Gut Microbiota can be described within the framework of ecological system, suggesting that such description could be helpful in the understanding of this interaction and in the discrimination of different Gut Microbiota.

We analysed GM sequencing data from [24], that include 5 different animals (one chicken, two swines and two cattle rumens). The stochastic neutral model proposed by Volkov for the coral-reefs and tropical trees ecosystems [22] was applied to the GM RSA and showed a good agreement with empirical data. An exception is found in the RSA tail, in which the model does not fit well the data. Such discrepancy implies a deviation from the purely neutral model, that anyway can be considered a good and simple approximation of the GM RSA. Moreover, the fact that this model works for such different ecosystems (coral-reefs, tropical forests and Gut Microbiota) emphasizes the universality of the RSA distribution and of the mechanisms that cause it, as suspected by many authors [18].

We showed that this simple neutral model, in which species are considered to be equivalent and species interaction is neglected, is also able to differentiate between the different animal species from which the Microbiota was sampled. In fact, the *S*/*b* parameters of animals belonging to the same species cluster together, while separate from those belonging to other species. This is evident if we plot *S*/*b* versus the similarity threshold used for OTU computation. The curves drawn by the *S*/*b* of different animals show different trends, while being alike for animals of the same species, and this means that different animals can be distinguished according to the influx parameter *S*, that has also a biological interpretation, being related to density dependence effects. For all samples *S*/*b* decreases at higher similarity thresholds. This indicates that the RSA extends from a Log-Series like to a bell-shaped Negative Binomial distribution when shifting from thinner to broader levels in the phylogenetic tree, a phenomenon analogous to the one observed for the coral-reefs and tropical trees ecosystems when local communities were assembled into meta-communities.

Finally, we showed that the biodiversity number *θ* derived by this neutral model increases with similarity and again differentiates between animals. This result is consistent with the biodiversity computed through the first and second order Hill’s numbers, with the difference that, while *H*_1_ and *H*_2_ simply relies on the fraction of individual per species, *θ* is based on a dynamical explanation of the ecosystem.

**Fig. 6:**
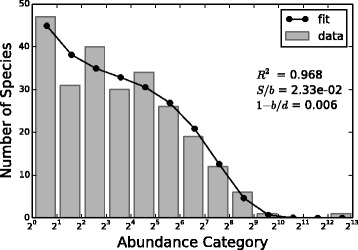
Human GM RSA and fit with Eq. 10. RSA of one human Gut Microbiota from [12] and fit with Eq. 10. OTUs were built with UCLUST with 95 *%* similarity threshold

The study of the animal GM is very useful both for the animal health and for the industry related to animals, but of course, as human beings, our main interest could be more on human health and human GM. We began to analyse some human data approved by the Cork Clinical Research Ethics Committee, from [12], following a similar procedure. Figure 6 shows the result for one human GM sample clustered with a 95 *%* similarity threshold. The model seems to work well also on human samples, suggesting that it could describe the GM of a wide range of organisms. In future, we will deepen the study of the human Microbiota RSA with the aim of building a predictive model, starting from the RSA distribution, that could be helpful in facing different inflammatory and metabolic diseases, that are, as previously outlined, closely related to the Microbiota composition. We surmise that the dynamic properties of the GM, caused by its ecological complexity and interactions with the host, can be modelled within the framework of stochastic population dynamics, including classical models such as predator-prey and ecological/microbial growth equations as species Lotka Volterra and Chemostat growth.
